# The Co-evolution of Honesty and Strategic Vigilance

**DOI:** 10.3389/fpsyg.2016.01503

**Published:** 2016-10-13

**Authors:** Christophe Heintz, Mia Karabegovic, Andras Molnar

**Affiliations:** ^1^Department of Cognitive Science, Central European UniversityBudapest, Hungary; ^2^Department of Social and Decision Sciences, Carnegie Mellon UniversityPittsburgh, PA, USA

**Keywords:** honesty, dishonesty, evolution, prosociality, social cognition, cooperation, partner choice

## Abstract

We hypothesize that when honesty is not motivated by selfish goals, it reveals social preferences that have evolved for convincing strategically vigilant partners that one is a person worth cooperating with. In particular, we explain how the patterns of dishonest behavior observed in recent experiments can be motivated by preferences for social and self-esteem. These preferences have evolved because they are adaptive in an environment where it is advantageous to be selected as a partner by others and where these others are strategically vigilant: they efficiently evaluate the expected benefit of cooperating with specific partners and attend to their intentions. We specify the adaptive value of strategic vigilance and preferences for social and self-esteem. We argue that evolved preferences for social and self-esteem are satisfied by applying mechanisms of strategic vigilance to one's own behavior. We further argue that such cognitive processes obviate the need for the evolution of preferences for fairness and social norm compliance.

## Introduction

### Honesty from an evolutionary perspective

In this paper, we consider honesty, dishonesty, and trust from an evolutionary perspective. Our main goal is to contribute to psychological theories about the patterns of dishonesty that have recently been documented in experimental economics. The main finding is that people tend to cheat when there are clear incentives to do so, but they cheat only by small margins. Ariely (2012) described this pattern as resulting from a “fudge factor.” We analyse how the fudge factor could itself result from human **evolved prosocial preferences**.^1^ Specifically, we ask: what is the **evolutionary function of the psychological traits** that grounds the observed patterns of behavior? The evolutionary perspective leads to question why and how such traits could be adaptive, and thus enables the formulation of hypotheses which need to be consistent with both evolutionary theory and empirical data.

The words honesty, truthfulness, and sincerity are often used interchangeably, thus limiting the scope of honesty to truth-telling. However, in the present paper we refer to the original and broader meaning of the word **honesty** as qualifying a disposition to act according to one's community's moral rules and to abide by its social norms (e.g., do not steal, do not lie, be generous). Saying that people are honest means that one approves of their social choices and attitudes toward rules of social interactions. Honesty is usually associated with bearing a cost: forgoing a potentially better, but dishonest, opportunity (e.g., refraining from cheating). In our day-to-day interactions, we start with the presumption that people will be honest and we are shocked when they are not. This might guide the investigation of honesty and dishonesty toward finding the cause of the latter, rather than the cause of the former. For political economy too, the temptation is to focus on the factors that foster dishonesty: uncovering them could guide political interventions aimed at reducing the associated costs. From an evolutionary perspective, however, the question is why people are honest at all. This is a puzzle for evolutionary psychology, because honesty leads to paying a direct opportunity cost. Often, when we say that someone is honest, we do not mean that she is rational and aware of the risk of punishment for misbehaving; rather, we mean that she accepts to pay the opportunity cost for the sake of complying to a certain rule of behavior that regulates social interactions. Honest behavior is thus characterized by choices where people forbear benefits so as to not deprive others from their own benefits, conceived as their due because of some rule. The preference that motivates honest behavior can therefore be interpreted as prosocial. **Prosocial preferences** stand in need of explanation for evolutionary theory because their adaptive value is not obvious. We argue that explaining how the prosocial preferences can have an adaptive value allows us to form hypotheses about the specific content of these preferences and the cognitive mechanisms on which they are likely to rely. A better understanding of why and when people are motivated to be honest can thus lead to a better grasp of the causes of dishonesty.

### Partner choice theory and the evolution of strategic vigilance

According to **partner choice theory** (see, e.g., Noë and Hammerstein, 1995; Barclay, 2013; Baumard et al., 2013), cooperative behavior and trust evolved in an environment with multiple opportunities for mutually beneficial cooperation. Seizing these opportunities, however, required being able to cooperate with individuals who make the cooperation worth for the self. How can one manage to cooperate with the partners who make the cooperative actions as beneficial as possible? First, one must be able to distinguish and select the **good cooperators**. A good cooperator is one who contributes a considerable amount of effort to a joint activity, and is efficient and competent. He thus contributes to the production of a large benefit. To make this cooperation beneficial for the self, it is also important that the partners let the self benefit from the product of cooperation. Good cooperators leave a sufficiently large share of the benefits to their partners, and do not cause additional cost by fighting over these benefits. Second, one must be selected for cooperative activities by good cooperators, so one must convince them that one is a good cooperator as well. Selection pressures therefore ensue for the evolution of cognitive capacities and psychological traits that: (1) discern who the good cooperators for given activities are, and (2) convince others that one is a good cooperator.

We hypothesize that humans have a set of cognitive capacities that allow them to predict, with sufficient reliability, whether cooperation with a potential partner will be worthwhile. Adequately choosing a partner for a cooperative activity requires assessing the competence of the potential partner for the task, their availability for doing the task and their willingness or intention to cooperate (Barclay, 2013). The latter requires vigilance because in the strategic context of partner choice, it is in the interest of others to convince us that they are good cooperators even if they are not. **Strategic vigilance** evaluates the expected value of engaging in a cooperative activity with potential partners in view of the selfish material interest they might have, their honesty, generosity, moral attitude and, more generally, their trustworthiness.^2^ A key feature of strategic vigilance consists in assessing potential partners' cooperative dispositions. Strategic vigilance enables one to avoid cooperating with partners who, although competent, will leave one worse off. Imagine a scenario in which you can go hunting with the best hunter in your community, but know he will keep most of the prey for himself. In this case, you are better off hunting with a less talented hunter who will share more. This is indeed what Bird and Power (2015) found in the Martu society: hunters who were generous and shared the most—but not who were the most skilled—were selected the most frequently for cooperative hunts.

**Glossary**

***Biological markets:*** situations where organisms can choose partners for interactions (e.g., choice of mating partners). Adaptive decision making will lead organisms to choose the best partner they can ‘afford’ for a given interaction (manage to convince to interact with them).

***Calculated Machiavellian strategy:*** the combination of a preference for material benefits and an understanding that, in some strategic situations, it pays off to invest in reputation.

***Evolutionary function of psychological mechanisms:*** what evolved psychological mechanisms, including preferences, do, that increases inclusive fitness, thus explaining why they have evolved.

***Evolved prosocial preferences:*** prosocial preferences that exist because they have an evolutionary function. Evolutionary theory warrants the assumption that such preferences will have long term positive effects on the organism's inclusive fitness even though they motivate choices with immediate costs.

***Honest choice:*** the term is commonly used to refer to social choices motivated by moral attitudes or guided by social norms. ‘Honest’ is used to qualify people who are judged to be desirable cooperators because of their character (rather than because of their skills). In this paper, we use the term in a specific way: we say that a choice is honest when one forbears benefits so as not to deprive others from benefits that are conceived as their due because of some rule. We use ‘honest choices’ to qualify choices where complying to the rule has an opportunity cost. Thus, honest choices are a specific kind of prosocial choices. For instance, most of the experimental results we review in this paper analyse choices to lie or tell the truth, when there is an incentive to lie. There is a rule of behavior that requests people not to lie, and lying leads to decreasing trust, which is a common good.

***Partner choice theory:*** a theory in evolutionary biology specifying selection pressures that would, in the human case, have influenced the evolution of social cognition and social preferences. According to this theory, people gained fitness by pairing with (i.e., choosing and being chosen by) the best partners for cooperating in mutually beneficial entreprises.

***Prosocial behavior:*** behavior that benefits others at some cost to the self. The cost and benefits considered are direct consequences of the behavior, but include opportunity costs. We argue that the long term consequences of such behavior are on average positive.

***Prosocial preferences:*** psychological mechanisms that produce motives for making prosocial choices. We use a mentalistic notion of preferences and refer to a psychological reality that determines choices.

***Proximate mechanisms:*** how evolved psychological mechanisms work.

In this paper, we assert that evolved prosocial preferences and calculated Machiavellism are two different proximal mechanisms whose evolutionary function is to manage reputation so as to do well in a market of potential cooperators.

***Strategic vigilance:*** we coined this term to refer to a set of capacities whose function is to predict when are potential partners likely to have cooperative intentions, and what can lead them to have such intentions. Capacities of strategic vigilance form a subset of capacities of social vigilance, which evaluate the expected payoffs of social interactions.

### Partner choice theory and the evolution of prosocial preferences

Once a form of strategic vigilance has evolved, a new selection pressure arises: one gains in fitness by convincing strategically vigilant others that one is a good cooperator. Doing so will motivate them to engage in cooperative activities that are beneficial for oneself. The stake, then, is to manage one's image and reputation: the beliefs that others hold about one's worth as a potential cooperator.

What are the types of behavior that can efficiently convince strategically vigilant others that one is a trustworthy cooperator; and what is the preference that will motivate people to adopt such behavior? Reputation management can be implemented by means of standard preferences for material benefits, combined with cognitive capacities that predict how behaviors affect reputation and how likely a good reputation will lead to higher material benefits. We can call this the **calculated Machiavellian strategy**, because it requires an evaluation of the costs and benefits of investing in reputation. When following this strategy, people will make prosocial choices only in the situations in which they predict that these choices will induce others to cooperate, and that the long-term material benefits will outweigh the cost of behaving prosocially. Humans have been shown to increase their future benefits in this way (Nowak and Sigmund, 1998; Wedekind and Milinski, 2000; Barclay and Willer, 2007). Mindreading—the human capacity to understand others' beliefs and desires—makes such computations feasible. This ability not only enables cooperation between non-kin (Barrett et al., 2010), but has also been shown to be a significant predictor of cooperativeness: people who have better mindreading skills show a stronger tendency to cooperate (Paal and Bereczkei, 2007). However, human psychology supplements calculated Machiavellian strategies with mechanisms that manage reputation without requiring the agent to conduct a deliberate analysis of expected costs and benefits (see Section The Function and Content of Prosocial Preferences). Such mechanisms can induce prosocial choices without the individual intending to derive long term benefits from these choices. For instance, one can desire to have a good reputation *per se* rather than as a means to derive long term material benefits. Concern for reputation can induce prosocial choices without the intention to obtain material benefits in the long term. Other such mechanisms, we will argue, include self-concept maintenance.

Partner choice theory leads to hypothesize that some of the human prosocial preferences evolved because they produce behaviors that efficiently convince others to choose us for self-beneficial cooperative activities (Sperber and Baumard, 2012). That humans have prosocial preferences can be observed in our day-to-day interactions with others: charitable giving, holding the door and other civilities are all expressions of prosocial dispositions. **Honest choices** (as we use the term in this paper) also fall into this category, because they imply *not* taking advantage of every opportunity to increase one's own payoff at the expense of someone's else.

How can honest, or in general, prosocial choices be a means of improving benefits to the self, since they are by definition not consciously intended to do so, and in fact have the opposite effect in the short-term? Even if not intended, the choices increase expected gains and contribute to fitness by maximizing the number of future cooperative opportunities—it is their **evolutionary function**. This is the ultimate explanation of the behavior, which specifies how it contributed to increasing the inclusive fitness of the behaving organism in the environment of evolutionary adaptedness. Proximate explanations, on the other hand, specify the actual mechanisms which produce the behavior and the conditions in which they do so. In the case of prosocial behavior, the *proximate* cause can include preferring fair outcomes, preferring to have a good reputation, preferring to maintain self-esteem, and skills and preferences for calculated Machiavellian strategies. These are **proximate mechanisms**.

### The function and content of prosocial preferences

We have a plausible hypothesis about the general evolutionary function of prosocial preferences, but still need to specify the selection pressures that lead to the evolution of such psychological traits. In particular, we need to specify why calculated Machiavellian strategies would not suffice as proximate mechanisms for reputation management. We can presume that calculated Machiavellian strategies were available before prosocial preferences evolved because the capacities they rely on are, in some form, available to non-human primates (see, e.g., Call and Tomasello, 2008). Furthermore, selection pressures for prosocial preferences can appear only if others are sufficiently strategically vigilant; and strategic vigilance exactly requires the ability to make reliable predictions about the future behavior of others. An account of the evolution of prosocial preferences therefore needs to answer the following question: what selective advantage can prosocial preferences provide when compared to a calculated Machiavellian strategy? One possibility is that prosocial preferences lead to making the adaptive choices a calculated Machiavellian strategy would recommend, but without paying the cost of computing the probability that the choice will indeed influence the audience's behavior in a way that sufficiently increases future benefits. However, given our dedicated skills to predicting others' behavior, sparing the cognitive cost of strategic computation can only be a small advantage, especially given that social preferences necessarily lead to making ‘mistakes.’ In view of their evolutionary function, prosocial preferences lead to false positives: decisions to act generously, while the expected consequences—in terms of material benefits—are not worth the cost. Another possible advantage of social preferences can be found if considered through the lens of error management (Haselton and Buss, 2000): while social preferences can lead to false positives, they also importantly reduce the number of false negatives. In that context, a false negative consists in failing to act prosocially because one underestimates the positive future consequences of the prosocial act for the self. For instance, one can incorrectly believe she is not being observed and decide to cheat, consequently being ostracized from her group. Mechanisms for decreasing the rate of false negatives can be heuristics that supplement the calculated Machiavellian strategy. For instance, choices are often sensitive to the presence of an audience: observability has been shown to be one of the primary factors which lead to increasing cooperation in real-world situations (for a recent review, see Kraft-Todd et al., 2015). However, people are not necessarily aware of this influence: the mere presence of photos of eyes has been shown to increase generosity and norm abidance in various settings, presumably because it triggers unconscious mechanisms for reputation management (Haley and Fessler, 2005; Bateson et al., 2006; for a meta-analysis of relevant constraints see Sparks and Barclay, 2013). Altruism can be prompted unconsciously by environmental cues that directly affect behavior. Prosocial preferences, however, are mechanisms that do not affect behavior directly, but do so via motivations which, in turn, affect planning and choice, and—eventually—behavior. They can trigger the conscious willingness to altruistically benefit others, or, equivalently, not to dishonestly exploit them.

Bounded rational agents (Simon, 1982) are unable to foresee many of the long term positive consequences of prosocial choices. Because of the preference for immediate gains, choices stemming from Machiavellian strategies are likely to miss many occasions to act prosocially for future benefits—i.e., to issue false negatives. This is especially plausible in a market of cooperators where people have to compete to be selected for cooperative activities. Moreover, this competition does not fully occur at the level of choice, but includes the level of the underlying motivations of the choices as well. This is because selectors are strategically vigilant; and know that someone who made an altruistic choice because of selfish intentions is less likely to cooperate in the future than someone who made an altruistic choice because of prosocial preferences (Karabegovic and Heintz, in preparation). In that context—where strategically vigilant agents take intentions into account—candidates for partnership have no choice but to mimic prosocial dispositions. Now, if the selectors' strategic vigilance is accurate enough, the best way to appear as someone who has prosocial preferences is to be someone who actually has such preferences. In the next section, we will argue that strategically vigilant others are likely to tell apart strategic motivations from genuinely prosocial ones.

Partner choice theory specifies the selection pressures for the evolution of prosocial preferences as follows:

- Multitude of beneficial cooperative opportunities.
- Seizing these opportunities requires convincing potential cooperators that one is a better cooperator than others.

Partner choice theorists have made the two above points clear, and this has enabled them to specify the function of prosocial dispositions. The specification of the selection pressures and evolutionary dynamics that operate on the market of cooperators provides ground for developing hypotheses about the most efficient means for matching with good partners. For instance, Baumard et al. (2013) have argued that a preference for fairness is such a means: it is an adaptive mechanism for partner choice. These analyses focused on the mechanism responsible for being selected. Little attention has been given to the other side of the evolutionary process: the mechanisms enabling the selection (but see Sperber and Baumard, 2012). However, it is equally important to understand the mechanisms that enable the selection of partners, because they form a crucial aspect of the selection pressures. We thus add a third point to the list above:

- The potential cooperators to be convinced are strategically vigilant.

What does it really take to convince strategically vigilant others that one is a good cooperator? Our claim is that dealing with this question might help explain the patterns of behavior that have not been sufficiently considered by partner choice theorists: patterns of generosity (Heintz et al., 2015), but also—the focus of this paper—patterns of dishonesty.

In this view, social preferences are mechanisms which motivate behavior that strategically vigilant others would value. An adaptive preference achieving that goal is a preference for *being* a good cooperator according to the potential judgments of strategically vigilant and worthy cooperators. To recover that judgment, one can put one's own strategic vigilance to work on one's own behavior. The experiments on dishonesty we review in the next section suggest exactly that: people want to think of themselves as good cooperators, and this enables them to cheat, but only a little. In the third section we go back to what we know about strategic vigilance, and in the last section, contrast our hypothesis with the preference for norm-abidance and preference for fair outcomes: two other preferences that could provide the evolved basis of prosocial dispositions.

## Proximate mechanisms of strategic vigilance

### How is strategic vigilance exercised?

Partner choice theory specifies a set of problems that our ancestors had to solve in order to benefit from cooperative ventures. We identified two subsets: first, they had to decide whether to enter a specific cooperative venture with a potential partner. This problem implies choosing with whom to cooperate among the available potential partners. Second, they had to maximize the overall value of beneficial cooperative opportunities. In a **biological market** (term coined by Noë and Hammerstein, 1994, 1995), this involves inducing the best potential partners to choose to cooperate with oneself. In a market of strategically vigilant others, it means convincing them that one is a partner worth cooperating with. The hypothesis of partner choice theorists is that having prosocial preferences is one means to do exactly that. We want to further investigate what it plausibly takes to convince strategically vigilant others that one is a good partner. In order to do that, in this section we review a set of studies about the proximate mechanisms of strategic vigilance. How are potential partners evaluated and how are predictions about their behavior formed? In that process, which aspects of potential partners' behavior are being especially attended to?

Strategic vigilance is most probably implemented by means of multiple cognitive mechanisms. These might include evolved capacities that allow people to detect potential cheaters, based on simple cues in a quick and efficient way, so that they can avoid unfruitful interactions with them (e.g., Cosmides and Tooby, 1992; Sugiyama et al., 2002; Petersen et al., 2012). Similarly, but independently from cheater detection, people might possess an evolved ability to identify prosocial and altruistic attitudes of others (Brown and Moore, 2000; Oda et al., 2006). However, incentives for prosocial or dishonest actions vary from context to context, and studies have shown that certain external factors, such as group membership or social distance, affect how prosocially people behave (e.g., Hoffman et al., 1996; Chen and Li, 2009; Engel, 2011). Furthermore, people are able to modulate their expectations of others' prosociality in view of these external factors (e.g., Goette et al., 2006).

Even though prosociality and dishonesty might be limited to the actual situation, the rich and blooming literature on moral judgments shows that people are able to make more abstract and more general moral evaluations of others (e.g., Haidt, 2007; Greene et al., 2009). This ability and tendency to make moral judgments is also tightly connected to strategic vigilance, because these judgments inform partner choice decisions.

There is ample evidence showing that people form social judgments in a way that is relevant for assessing potential partners, e.g., people who are honest or dishonest, nice or nasty, generous or selfish, competent, or incompetent, and so on. Two dimensions which reflect this evolutionary pressure emerge consistently in the literature on social perception: warmth (which encompasses the honest-dishonest, nice-nasty, and generous-selfish dichotomies) and competence (Fiske et al., 2007). How people predict behaviors in a cooperative context is also relevant to understanding strategic vigilance. Several studies have investigated whether participants can predict their partner's decision in experimental settings. When people can observe their partner's past choices, they can predict future choices by using fairly simple strategies such as reinforcement learning (see e.g., Erev and Roth, 1998). However, there is convincing evidence that people can predict others' choices even without observing their past behavior. In prisoner's dilemma games participants are able to predict their partner's choice with an accuracy rate significantly above chance if they are allowed to meet in person and communicate before decision-making (Dawes et al., 1977; Frank et al., 1993; Brosig, 2002). Similar results have been obtained for high-stake, real life settings as well: third-party observers were able to predict game show participants' choices at an accuracy above chance (Belot et al., 2012). Other studies have shown that people can predict others' choices without having any prior interaction—for instance, when they were presented a single photo of the partner (Yamagishi et al., 2003; Eckel and Petrie, 2011). Verplaetse et al. (2007) showed that even third-party observers are able to distinguish cheaters from cooperators, based on a single photo that was taken of the target person at the moment of decision-making.

Eventually, one would like to know how people actually choose their partners. There are only a few experiments and field studies which examine such strategies (e.g., Delton and Robertson, 2012; Lyle and Smith, 2014; Bird and Power, 2015). Mindreading and moral judgments are definitively central to deciding when and with whom to cooperate. However, in this section we will focus on the ascription and evaluation of intentions of potential partners.

We argue that one important way strategic vigilance is exercised is by representing and evaluating others' intentions. In the next section, we will review evidence showing that the judgment about the honesty of a choice depends on the underlying intentions the choice reveals. Thus, inferences about underlying intentions are at the core of both strategic vigilance and prosocial choices. We will argue that honesty is driven by exercising strategic vigilance to one's own choice.

### Computing intentions gives predictive power

We gather information about others' past behavior and the outcome of their actions, either through direct experience or through indirect channels, such as reputation systems. How do we infer future behavior on the basis of these data? In particular, how do we predict potential partners' behavior in a given cooperative venture? The main difficulty is that the willingness to cooperate depends on unobservable mental factors which can only be inferred from instances of past actions and the context in which they transpired.

An outcome-based assessment of potential partners calculates the benefits derived from past interactions with a partner, and leads to the conclusion that the expected benefits of future interactions are similar. Several variations can be implemented: for instance, averaging the payoffs of past cooperative ventures with a given person and assuming that the future benefit will be around this average, or giving larger weights to more recent payoffs (e.g., reinforcement learning: Erev and Roth, 1998; experience-weighted attraction learning: Camerer and Ho, 1999). The relevant aspect of such processes, however, is that they do not compute partners' intentions. We argue below, first, that predicting behavior on the basis of inferred underlying intentions allows for more accurate predictions, and second, that humans actually do compute and evaluate the intentions of potential partners.

Outcome-based inferential processes are limited on two fronts. For one thing, humans interact on a wide range of tasks, but outcome-based cognitive processes do not allow making reliable inferences from behavior observed in one type of task to another. For instance, Jenny might be a competent football player, yet an enthusiastic but poor writer. Therefore, if co-authoring with Jenny is not worthwhile, we cannot conclude that being on her football team would be similarly painful. In order to evaluate across tasks, people distinguish underlying intentions, preferences and task-specific capacities. For another thing, outcome-based cognitive processes do not enable making reliable predictions when the context changes, even if the task remains the same. In particular, the partner's incentives and situational constraints can change from observed past choices to the present situation. For instance, the probability of meeting and having an interaction with a particular partner might drastically decrease and approximate zero. In that case, the expected value of signaling prosociality diminishes because there will be no further opportunities for mutually beneficial cooperation—the incentives have changed. In such cases, past benefits of cooperation become a poor predictor because the context has changed with regard to the future.

Outcomes in and of themselves do not reflect the intention of the agent. For instance, a selfish agent can help and be generous, but only to rip off the naive agent at the most opportune occasion down the line. Similarly, a helpful agent can simply fail to deliver benefits because of an unlucky set of circumstances. Computing intentions allows for the integration of highly relevant information about the context in which the cooperative action took place. Contextual aspects of great significance include the incentives that the partner faced and the affordances she benefitted from.

How, then, can we predict behavior on the basis of past intentions? One hypothesis is that people infer others' intentions from their actions and then further infer underlying dispositions and skills from intentions and context. In particular, people attribute social preferences to others that account for motives that are not self-interested. It is these inferred social preferences that eventually matter when it comes to forming beliefs about cooperativeness. Such beliefs allow people to make predictions about future cooperative choices in diverse contexts. The processes include:

1. Inferring intentions from outcomes and contextual information. In particular, when someone makes a choice that has beneficial consequences, we can infer whether these consequences were side effects or whether they were intended.
2. Making inferences from the content of intentions in one instance to the individual willingness to cooperate in other contexts. One possible way to do this is to infer personality traits of the agent. For instance, if someone has the intention to make altruistic choices, then we think of her as an altruistic person. On the basis of this disposition, one can make predictions about future choices in new contexts and for new problems. Other types of inferences might be at work.

Computing others' intentions enables integrating many contextual information for assessing how cooperative one is likely to be in the future. We have noted that this contextual information is relevant to strategic vigilance because an outcome can be beneficial or detrimental due to contextual aspects that are unlikely to occur again. Recovering underlying intentions teases apart multiple factors that lead to the outcome: competence, environmental opportunities and, possibly, interests in specific cooperative ventures, as well as other mental states.

We have now pointed out the increase in accuracy which computing intentions might provide, compared to outcome-only-based inferences. In the next sub-section, we review empirical evidence showing that human strategic vigilance is a set of implemented processes that indeed rely on inferred intentions.

### Evaluating intentions: empirical evidence

With 40 years of psychology on mind-reading,^3^ there is plenty of evidence suggesting that people can successfully infer others' intentions, even if these conflict with the observable outcomes (e.g., Cushman, 2008; Ames and Fiske, 2015; Rand et al., 2015). Even young infants and children form expectations of agents' behavior that rely on inferred intentions (e.g., Gergely et al., 1995; Meltzoff, 1995; Sutter, 2007).

Intentions, having been computed, are also taken into consideration in moral evaluation. Moral judgments, indeed, are made on the basis of both unobservable mental states (intentions, beliefs, and desires) and observable physical states (physical constraints, exerted effort, and realized consequences, Cushman, 2015). In particular, people distinguish situations where the good or bad outcome was intended or not, and evaluate the acting agent's personality accordingly (Tooby et al., 2006; Cushman, 2008). In experimental economic games, participants appreciate others' good intentions, despite their failure to contribute (Rand et al., 2015). There is some evidence that even preverbal infants judge agents in view of their intentions rather than on outcome alone: a study by Hamlin (2013) showed that 8-month-old infants prefer puppets who, despite their helping efforts, fail to help another, over puppets who try to hinder another in distress, but accidentally help. Although it is still debated whether infants this young really prefer helpers to hinderers (see Salvadori et al., 2015), there is a consensus that preschool-aged children can already make sophisticated moral judgments when intentions and outcomes are not necessarily aligned (Leslie et al., 2006; Vaish et al., 2010).

We do not claim that people are completely unaffected by outcomes when they evaluate others' cooperativeness or infer other social dispositions. What we emphasize is that people are able to infer underlying intentions, *in addition* to evaluating others based on the observed outcomes of their actions. Therefore, the mere existence of the “outcome bias” (i.e., when moral judgments are affected by observed outcomes, see Gino et al., 2009b) does not contradict this proposed aspect of strategic vigilance; that is, people infer and take into account intentions when evaluating potential partners. In a series of cross-cultural studies, Barrett et al. (2016) revealed that moral judgments are universally influenced by intentions, even though societies can give a larger role to the outcomes when evaluating an agent's actions—possibly because of cultural beliefs that people's intentions are hard to guess (Barrett et al., 2016).

One relevant piece of information for evaluating intentions is the observed effort an agent makes in order to achieve a certain outcome, or the cost that an agent incurs when she interacts with others. All else being equal, the more effortful an unselfish action is in terms of energy, time or monetary cost, the more effectively it signals that one is helpful and creates the impression of prosociality. This is indeed what Delton and Robertson (2012) found in a series of studies in hypothetical food foraging scenarios. Their participants rated people who incurred a larger cost, but provided only small benefits more positively than people who provided large benefits but incurred a minimal cost. This result supports the assertion that adults make sophisticated judgments about others' cooperativeness, and take into account intentions, by looking at the incurred costs and exerted effort as well as the outcomes. Following the same line of argument, ethnographic studies in pre-industrial societies have documented that an individual's cooperativeness is not simply judged by the amount of goods or services he provides, rather it is usually inferred from the effort the individual makes, or the cost he incurs when taking part in a collective activity (Price, 2006; Lyle and Smith, 2014; Bird and Power, 2015). The ability to conditionally reciprocate others' good intentions with good actions also begins to emerge early in childhood. A study by Dunfield et al. (2013) showed that children, by 3 years of age, can already identify situations when a communicator is helpful, and in turn, selectively cooperative with her. By the age of 7, children already anticipate that others will infer intentions from their (i.e., the children's own) actions, and are motivated to maintain a positive image. For instance, Shaw et al. (2016) show that 7–8 years old children can already apply sophisticated, context-dependent strategies when they decide about the allocation of goods between themselves and others.

Another key element for strategic vigilance is assessing the credibility of trustworthiness signals (Brosig, 2002; Henrich, 2009). Rockenbach and Milinski (2011) found that people are able to identify situations in which they have to be more vigilant because the potential benefits for dishonest signaling are high. The importance of this ability is reflected in the results which show that human strategic thinking becomes sophisticated quite rapidly, allowing us to detect manipulative intentions early in childhood (Ayal and Gino, 2011). By the age of 4, children are able to distinguish others' accidental errors from intentionally deceptive actions, and can therefore identify agents who are more likely to deceive them in a certain context (Mascaro and Sperber, 2009). By the age of 7, children can already anticipate deceptive moves in complex strategic settings with novel partners (Sher et al., 2014), and make subtle evaluations about trustworthiness when honesty conflicts with benevolence (Xu et al., 2013).

Cognitive processes enabling adaptive choices of partners for cooperative activities are far more complex than a weighting of past benefits gained from cooperation. These processes provide the means for integrating a lot of relevant information from the context in addition to information about the outcome of past cooperative activities. We have argued that one way to do that is to infer and evaluate the underlying intentions of choices in strategic situations. Strategic vigilance, we conclude, pays special attention to the intentions that generate the observed behavior and evaluates what these intentions reveal about the cooperative dispositions of the potential partner (his moral and prosocial preferences, for instance). We will now point to a striking parallel with choices putting one's own honesty at stake: these choices are such that the underlying intentions cannot be interpreted as uncooperative. It suggests that these choices are driven by putting one's own strategic vigilance at work on one's own behavior.

## Adaptive preferences cause patterns of honesty

### Experimental evidence on dishonesty

Recent experimental studies about dishonesty specify the limiting conditions that reveal the trade-off between taking as much as possible from an interaction and the desire to act as a worthwhile cooperator (Mazar et al., 2008). In a series of studies examining the propensity to cheat researchers asked participants to roll dice and report the result. Participants had an incentive to lie because certain numbers had higher payoffs than others. Furthermore, participants were ensured that nobody but themselves would know what the number really was (Fischbacher and Föllmi-Heusi, 2013). The finding is that people do lie, but tend to report a credible result that does not depart from the true numbers too much. For instance when they could maximize their payoff by rolling a 6, they did not always report 6, but still lied a few times. Only around 2.5% of participants lied to maximize their gains, regardless of the actual outcome observed (Shalvi et al., 2011).

These patterns of behavior suggest that people are motivated to uphold an image of themselves as honest cooperators: they want to signal that they are willing to incur costs or forgo potential benefits by ‘playing by the rules.’ And indeed, when people cheat only a bit, within the plausible limits, the act of cheating is hardly detected by others. In one of their follow-up studies, Fischbacher and Föllmi-Heusi (2013) investigated others' guesses about the distribution of die-rolls and found that 61% of observers unfamiliar with the paradigm would not detect partial lying. A similar pattern of lying emerged in the matrix experimental paradigm (e.g., Mazar et al., 2008; Gino et al., 2013), in which participants reported their performance on math-related tasks, and were incentivized to report a good performance. Participants tended to report solving more problems than they actually solved, yet they increased the true number only slightly.

Strategies in these experiments rarely maximize monetary payoff. Rather, the most common strategy consists in lying to *increase* monetary payoff, but not to the extent that could be easily detected. Since the reports are bent only inasmuch to remain plausible, people using this strategy have to take into account the capacity of others to detect lies: therefore the strategy is adapted to strategically vigilant observers. However, in the studies described above, such strategies were used in spite of the fact that being thought of as a blatant liar had no consequences in the experimental context where anonymity is credibly implemented. A straightforward explanation for the adoption of this strategy is that people unconditionally care about the opinion of others to some extent, regardless of the future consequences (Bénabou and Tirole, 2006; Ariely et al., 2009). Furthermore, decisions aimed at maintaining a good image are informed by the understanding that others have limited information about the context, yet are strategically vigilant—lying is possible, yet constrained by plausibility. For example, participants in Shalvi et al.'s (2011) study presumably perceived the desirable outcome as more likely to happen—and thus the lie as more plausible—than when they did not observe a die-roll with a high result.

Some strategies, however, cannot be explained by a preference for being esteemed by others: the maintenance of one's self-concept (Mazar et al., 2008) seems to play a role as well. Evidence for this claim comes from experiments using the protocols described above and manipulating how easily participants can justify their actions to themselves. The prevalence of dishonest behavior decreases when moral standards are made salient and increases when potential justifications are easily available. More precisely, factors that modulate the rate of dishonest choices include in- and out-group membership of the agents involved (Gino et al., 2009a), commitment, moral reminders (Mazar et al., 2008), and whether or not the self-serving lie could be plausibly justified as a mistake (Pittarello et al., 2015). In general, participants misreport more when they can present misreporting as resulting from an intention that is *not* uncooperative, like misperception of the number on the die rather than an intention to deceive. Similarly, misreporting guided by the intention to help potential cooperators such as group members is not seen as uncooperative: cheating increases significantly when it benefits others rather than the decision maker alone, wherein the presence of other beneficiaries decreases feelings of guilt and negative ratings of one's morality (Gino et al., 2013).^4^

### Psychological mechanisms and their adaptive value

The theory of self-concept maintenance, proposed by Mazar, Ariely and their colleagues is meant to account for the above findings. It asserts that what matters to people is whether the choices fall squarely in place with other clearly immoral actions or can be interpreted in a self-serving way as being moral. In the latter case, it is possible to reap the material benefits of dishonest choice, without having to update one's self-concept in the negative direction; a condition referred to as “relative malleability” (Mazar et al., 2008; p. 634). It is possible to categorize one's own choices as moral only up to a certain extent, and some choices inevitably lead to revising one's self-concept. The central claim of the self-concept maintenance theory is that negatively updating the self-concept is aversive, hence dishonesty will be more prevalent in cases where updating can be avoided. Similarly, attention to moral standards makes dishonest actions more salient in comparison to one's internalized beliefs about what is desirable or valued, and thus more likely to influence the self-concept. We extend the above psychological analysis with an evolutionary perspective. How can self-esteem or maintenance of a moral self-concept be an adaptive preference?

Self-concept maintenance adaptively is adaptive because it improves the performance of calculated Machiavellian strategy for reputation management. Because of human cognitive boundedness, people are limited in their ability to simultaneously track the multitude of beliefs that potential partners might have about them. Furthermore, people do not have the capacity to continuously and separately update all of these individual opinions in view of their choices. However, people can approximate others' opinions by applying their strategic vigilance to their behavior, thereby constructing and maintaining a self-image. When combined with a preference for self-esteem, this produces a willingness to behave as a good cooperator according to the standards of other worthwhile potential cooperators, yet without actually tracking their opinions. A desire for maintaining high self-esteem is a reliable cognitive mechanism for maintaining a good reputation (see Kurzban, 2012).

It has also been argued that if one believes oneself to be moral, it will be easier to convince others that one *is* moral (Trivers, 1985). Frank (1988) argued that having a genuine concern for one's moral standing is a reliable means for convincing others that one is a good potential cooperator. By contrast, alternating between self-serving dishonesty and prosocial signaling is likely to produce false negatives, cases where one chooses self-serving dishonesty without realizing its detrimental effect on reputation. Frank's hypothesis is justified because potential partners' strategic vigilance prevents one from gaming their value as a potential partner. Selfish intentions are likely to be recognized as such, and pro-social intentions are likely to be valued more, because they provide some evidence that the potential partner will not seize all contextual opportunities to increase her selfish gain at one's own expense.

Monitoring self-image is a reliable process for a bounded Machiavellian agent to maintain a positive reputation. Indeed, studies on dishonesty show that it is possible to maintain self-esteem while reaping as much of the material benefits as possible, because the motives for making prosocial choices are based on justifiability (Shalvi et al., 2015). This dependence on justifiability shows that people are prone to internally negotiate what it takes to be a good cooperator, and that this negotiation is done with constraints set by strategic vigilance. Selfish but justifiable courses of actions are consequently taken: this is adaptive because it does not affect reputation, while increases direct fitness.

The adaptive value of preferences for positive reputation and self-esteem also lies in the similarity between the choices that these preferences motivate and the choices that convince observers that the chooser is a desirable partner. In the case of a preference for positive reputation, the similarity is a consequence of an accurate understanding of what others value, and what behavior is likely to convince them that one is a good cooperator. In the case of the preference for self-esteem, the similarity is a consequence of evaluating one's own behavior with the same means, the same strategic vigilance, as observers would. Generalizing on that point, we predict that people will systematically build a self-image that appeals to the most worthy and accessible cooperators in their environment, and care less about the values of agents they deem to be worse cooperators. This process makes self-concept maintenance a proximal mechanism for reputation management that is flexible to the specific values of one's community.

Furthermore, prosocial preferences driven by the desire to maintain a good image are adaptive when they lead one to find the best trade-off between investment in reputation and immediate gains. This adaptive trade-off is best illustrated with the phenomenon of moral licensing, wherein a good deed can increase the likelihood of subsequent immoral behavior (for a review, see Merritt et al., 2010). In strategic vigilance terms, if one has accumulated ample proof of being an honest cooperator, she can allow herself to upset the balance by a self-serving action without the worry of seriously endangering self- and social image. The effect goes both ways: when primed by writing a negative story about themselves, people are more likely to donate to charity as opposed to after writing a positive or neutral story (Sachdeva et al., 2009). Similarly, studies in impression management have shown effects such as generalized image repair (Baumeister, 1982) which demonstrate that being alerted to one's negative reputation in a community elicits more instances of prosocial behavior (Steele, 1975).

The list of adaptive features of self-concept maintenance therefore includes the following aspects:

1. It decreases the false negatives that a calculated Machiavellian strategy and preference for maintaining a good reputation would produce: self-concept maintenance often motivates making the choices that one would make if he knew he was observed.
2. It convinces strategically vigilant others that one is a desirable partner better than selfish preferences and preference for maintaining a good reputation. This is because strategically vigilant others are very competent at recovering underlying intentions, and because selfish preferences and preferences for maintaining a good reputation are less likely to motivate the partner's prosocial choices across different contexts.
3. It motivates making choices that are valued by desirable partners and do not motivate prosocial choices that they are not likely to value. This is because:
  - The values of desirable partners are more likely to be internalized than the values of inaccessible and undesirable partners.
  - The opinion of desirable partners are reliably simulated in context by using one's own strategic vigilance.

Points 1 and 2 show that self-concept maintenance is a mechanism that improves the likelihood of being chosen by desirable partners for mutually beneficial enterprises. It improves this likelihood over self-interest seeking with guile and over interest in positive reputation. Point 3 shows that self-concept maintenance will not motivate making costly prosocial choices that are unlikely to increase one's value as a partner among desirable partners. In the next section, we contrast this adaptive feature with the features of evolved preferences for norm abidance and fairness.

## Conclusion

Predicting the future cooperativeness of potential partners is an inferential task based on the limited information provided by past behavior, observed in context. In particular, the desirable traits of potential cooperators are relatively opaque, especially with regard to their interest in cooperating in specific ventures, which depends on their social preferences, their immediate cost of cooperating, the expected cost of negative reputation and other factors. This has important consequences for the evolution of both strategic vigilance and social preferences. Strategic vigilance requires powerful inferential mechanisms whose evolved functions include the ability to estimate with whom it would be better to cooperate in the future. The hypothesis that human cognition evolved largely for dealing with social life can thus be enriched with a specification of the cognitive tasks which, when solved, make cooperation advantageous. Cooperation, rather than competition alone, would then provide selection pressures for the evolution of social cognitive skills (Moll and Tomasello, 2007).

In a biological market, the adaptive answer to strategically vigilant others is to anticipate their inferences about one's own behavior. We have seen that preferences for maintaining a good image in the eyes of others (reputation) and in one's own eyes (self-image) do just that. Are such evolved preferences complemented with evolved distributive preferences and/or a preference for abiding by the social norms? Let us consider them in turn.

### Norm abidance

Evaluations of honesty usually assess compliance with agreed-upon rules of action, whether these rules are explicit or not. Mazar et al.'s (2008) cognitive account of dishonesty assumes that there is a preference for a good self-image, that can be maintained by abidance to certain internalized moral norms or standards—such as ‘do not lie.’ They then make the hypothesis that this preference is supplemented with a capacity to muddle the boundaries of the class of actions that count as moral rule following. This further hypothesis extends the standard account which explains honest and dishonest choices as resulting from maximizing the utility, derived from either norm compliance or from selfish material gains. In this framework, people can choose to be dishonest when the temptation is high and/or when they have weak preferences for being honest. It leads to the prediction that deciding to break a moral norm will lead one to go ‘all the way’ to maximize material benefits, which has been refuted by experimental results showing that people often choose to cheat a little or lie by a little margin only. Assessing honesty is very different from a binary classification of actions (complied or not to the social norm), usually leaving plenty of wiggle room.

In particular, we have emphasized that a lot of interpretative work is being done: interpreting how to apply a rule in a given context and inferring the underlying intentions from actions. Ethnographic studies of moral behavior likewise show that moral norms are subject to diverse interpretations depending on the context and the interests of the people involved (e.g., Humphrey, 1997). In certain contexts, norms might conflict with each other (e.g., equality vs. effort-based distribution of goods), and people might ‘cherry-pick’ among available norms in a self-serving way. For example, when the equal allocation of goods provides more benefits, people tend to evaluate it as more morally justified, compared to the case in which they would receive more from an effort-based (unequal) allocation (Cappelen et al., 2014; DeScioli et al., 2014). This is not to say that anything goes: the ‘chosen’ norm and the corresponding action have to be adequately justifiable in the given context in order to convince strategically vigilant others that one is worth cooperating with.

There are quite a few factors that determine whether and how a rule of behavior will impinge on choices (Bicchieri, 2006). These factors include the content of the rule (e.g., one is reluctant to abide by a disgusting rule), the origin of the rule (e.g., it was dictated by a malevolent person), what one thinks others think of the rule (e.g., nobody thinks it is important to pay for tram tickets), as well as assessments of the costs and benefits of abiding by the rule. How are these factors combined in decision making? For instance, Gneezy (2005) investigated how the consequences of lying are evaluated. Mazar et al. (2008) do not specify which cognitive processes allow for muddling moral categories. Partner choice theory, together with the observations we made on strategic vigilance, lead to the hypothesis that people have evolved mechanisms that use agreed upon or social rules of behavior as source of information for what it takes to be a good cooperator in the eyes of potential partners. They have a good sense of what is expected of them, and these expectations are constantly informed and updated by communication (e.g., public debates about what should be allowed, legislation) and observations of the consequences of others' behavior (e.g., precedents, salience of actions, punishments and rewards). Mechanisms of strategic vigilance evaluate what can be expected from an interaction on the basis of implicit or explicit contextual agreements or cultural norms and, on the same basis, assess the justifiability of partners' potential expectations. It is during this process that one can find wiggle room allowing more selfish behavior. Still, rules or social norms *do* guide social behavior and foster prosocial choice, because ignoring them would lead to disappointing one's partner: a consequence people would prefer to avoid (Heintz et al., 2015). If one follows this thread of reasoning, it is the potential partners' expectations—whose content is influenced by the rule of behavior—that have a motivating power; not the social rule itself.

We have argued that preferences which motivate honesty are geared toward managing what others might think of oneself. In particular, one wants to be thought of as a good cooperator and also think of oneself as such. The behavior indicative of being a good cooperator is highly dependent on contextual factors as well as beliefs about partners' expectations that are informed by social norms. The evolutionary story that we advocate, based on partner choice theory, leads to a specific measure of adaptiveness that informs psychological theories. It can be contrasted with another evolutionary story of prosocial behavior and norm abidance: some social scientists (e.g., Richerson et al., 2016) have argued that people have a biologically evolved preference to abide by social norms and to do as the majority does. Because groups with prosocial social norms will do better than groups with non-prosocial norms, prosocial norms will thrive and people will end up preferring to abide by prosocial norms. This explains prosocial preferences as resulting from a preference to abide by (and enforce) social norms. But why would we then observe the small misdemeanors reviewed above? A *post-hoc* hypothesis could state that the processes interpreting social norms in context enable muddling them. Our evolutionary story, on the other hand, places the selection pressures elsewhere and leads to hypothesizing that norm abidance is a consequence of preferences that have evolved for reputation management. A comparison of the plausibility of each of those two theories is beyond the scope of this paper. Here, we have only described a consistent psychological and evolutionary theory of the patterns of honesty, which we believe to be a serious rival to cultural group selection theory explanations.

### Distributive preferences

Relying on partner choice theory, rather than cultural group selection theory, Baumard et al. (2013) have made hypotheses about the proximal mechanisms that evolved for impression management, which lead to making prosocial choices. We have argued that impression management for partner choice is done via three mechanisms: calculated Machiavellian strategies, a preference for maintaining a good image in the eyes of others, and a preference for having positive self-esteem. But for Baumard et al. (2013), impression management is achieved thanks to an evolved preference for fair outcomes. Has such a preference truly evolved to supplement the three mechanisms mentioned? There are two reasons to think that this is not the case.

First, an evolved preference for fair distributions would increase the number of false positives (making an altruistic choice which does not add to one's reputation). In many cases, choosing a fair distribution might overshoot, making an unnecessary sacrifice, and letting others benefit more than would be useful. For instance, if what is deemed honest by the members of one's community is to give no more than one third of the hunted game to a low-born hunting partner, then why would one give more than that? It might result in a distribution of costs and benefits that is directly advantageous to the high-born hunter, but in certain market conditions, this kind of practices can perdure. Of course, the economic dynamics of the market of co-hunters might, in some conditions, lead to the cultural evolution of fairer social norms, but the fair choices would then result from cultural evolution rather than directly from an evolved preference for fairness. One can increase immediate benefits and still be a good potential partner if others are satisfied with little or if it is socially acceptable to take more for oneself. Consequently, the means for maintaining a good reputation is to act on the basis of what others think is fair, which is partly determined by cultural traditions. In that perspective, the theory of evolved preferences for fair outcomes paints too rosy a picture of social life: assuming that people already have evolved intuitions about fair distributions and honest behavior gives too little importance to the social dynamics that spread cultural beliefs about what is fair and honest. By contrast, we predict that in a human market of cooperators, these beliefs are at stake. When a member of a community has a good grasp of what others think is fair and honest and has ‘internalized’ these beliefs, then preferences for fairness and norm abidance are redundant in the presence of preferences for good reputation and self-esteem.

The features of the preferences for others' and self-esteem which make it possible to reduce the cost of maintaining a good reputation are listed in Section Psychological Mechanisms and their Adaptive Value; to reiterate: the choices that would disappoint desirable partners are avoided, but opportunities to make self-serving choices that are unlikely to have a negative effect on reputation are promptly seized. Preferences for others' esteem and self-esteem are based on how choices can be justified. They rely on strategic vigilance put to work, this time, for monitoring the opinions of potential partners. It is unlikely that a preference for fair outcomes would significantly reduce the number of false negatives (failing to make an altruistic choice that would have importantly improved reputation) over preferences for others' esteem and self-esteem. We conclude that an evolved preference for fairness would not increase fitness derived from having a good reputation, yet, it would decrease fitness derived from making self-interested choices. It is therefore not obvious that a selection pressure existed for the evolution of a preference for fair distributions to supplement preferences for others' esteem and self-esteem.

The second reason to be skeptical about the existence of this type of preference is that it makes it difficult to account for a range of empirical data which show that people prefer justifiable self-serving choices rather than fair distributions. For instance, when they are rewarded for performance, it is unfair that the participants who misreport get more money than those who perform well, but do not misreport. Such data could be explained by saying that the preference for fair distributions, in some individuals, is less pronounced, and that the temptation to misreport is strong. But this does not account for the specific factors that modulate the rate of misreporting: justifiability in the eyes of potential observers and the self. Some experiments reviewed above provide evidence that manipulating the justifications available has an effect on rule compliance and prosociality even when the content of the social rule or potential distributions remain the same. The theory of evolved preferences for fair outcomes still needs to explain how such data can be accounted for. Similarly, Heintz et al. (2015) reviewed a set of experiments showing that, when participants are asked to make a monetary transfer, they are prompt to make unfair transfers when these will not disappoint their partners. For instance, participants will often choose means to decrease their partners' expectations from an interaction over sacrificing their benefits for a fair distribution (see, e.g., Dana et al., 2006; Hauge, 2016). Such situations reveal that what matters to people is to be able to justify their choices to their partners (and themselves), rather than the distribution of costs and benefits *per se*.

Recent theories explaining how partner choice can give rise to adaptive mechanisms that motivate making prosocial choices provide valuable alternatives over competitive theories of human prosociality, such as cultural group selection theory. Yet, partner choice theorists have given too little attention to the specifics of strategic vigilance as constitutive of the selection pressures for the evolution of mechanisms motivating prosocial choices. We speculate that mechanisms for reputation management and strategic vigilance co-evolved in an arms-race: mechanisms for convincing others of one's cooperative value evolved to outperform mechanisms of strategic vigilance by paying as little a cost as possible for cooperation, while taking as much as possible from its benefits, and mechanisms of strategic vigilance evolved to prevent gaming and select the truly best cooperative option. There are several competing strategies that allow having a good reputation. Among these strategies, some are more efficient than others: they achieve the goal of having a good reputation while keeping the cost from generous giving or the opportunity cost of honesty lower. In our opinion, a combination of preferences for others' and self-esteem produces the optimal balance of the latter, as well as providing a plausible evolutionary-flavored account of the recent findings from experimental economics.

## Author contributions

CH is the main author, MK and AM contributed equally.

## Funding

The research leading to these results has received funding from the European Research Council under the European Union's Seventh Framework Programme (FP7/2007-2013)/ERC grant agreement n 609819.

### Conflict of interest statement

The authors declare that the research was conducted in the absence of any commercial or financial relationships that could be construed as a potential conflict of interest.

## Footnotes

^1^Expressions in bold are explained in the glossary.

^2^Note that we are referring here to economic activities, the evolution of epistemic trust is another yet very similar story (see Sperber et al., 2010).

^3^Mind-reading is also called Theory of Mind, or mentalizing. The milestones that are often thought as the origin of the surge of research on mind-reading are Dennett (1978) and Premack and Woodruff (1978), which focused on attribution of false beliefs.

^4^The increase of lying when it leads to benefiting other people could be accounted for by a preference for reputation as well, i.e. the people who benefit from one's lie might hold the liar in higher regard. The preference for self-esteem and the preference for having a good reputation are tightly interconnected, but it is worth to analyse the effect of each separately.
